# Recent advances in understanding Lynch syndrome

**DOI:** 10.12688/f1000research.9654.1

**Published:** 2016-12-21

**Authors:** Sherief Shawki, Matthew F. Kalady

**Affiliations:** 1 Department of Colorectal Surgery, Digestive Disease and Surgery Institute, Cleveland Clinic, Cleveland, Ohio, USA

**Keywords:** hereditary colorectal cancer, CRC, Lynch-Like syndrome, Tumour Lynch

## Abstract

Colorectal cancer affects about 4.4% of the population and is a leading cause of cancer-related death in the United States. Approximately 10% to 20% of cases occur within a familial pattern, and Lynch syndrome is the most common hereditary colorectal cancer syndrome. Lynch syndrome is a hereditary predisposition to forming colorectal and extracolonic cancers, caused by a germline mutation in one of the DNA mismatch repair genes. Identifying at-risk patients and making a correct diagnosis are the keys to successful screening and interventions which will decrease formation of and death from cancers. Knowledge of the genetics and the natural history of Lynch syndrome has continued to be uncovered in recent years, leading to a better grasp on how these patients and their families should be managed. Recent developments include the approach to diagnostic testing, more precise definitions of the syndrome and risk stratification based on gene mutations, surgical decision-making, and chemoprevention.

## Introduction

Lynch syndrome (LS) is a genetic predisposition to developing colorectal cancer (CRC) and extracolonic cancer, caused by an inherited deleterious germline pathogenic variant in one of the mismatch repair (MMR) genes or *EPCAM*. Responsible for approximately 3% of all CRC cases, LS is the most common hereditary CRC syndrome. Affected individuals with LS tend to develop cancers at a young age and also have higher incidences of synchronous and metachronous cancers. Multiple organ systems are at risk, including the colon, rectum, uterus, ovaries, stomach, small bowel, urinary tract, pancreas, and skin. CRC presents the highest threat, and lifetime risks are 53–69% and 33–52% for males and females, respectively ^1^. The identification of Lynch kindreds followed by appropriate surveillance and management is critical to reduce the formation of cancers and mortality associated with the disease. For example, colonoscopy reduces both the incidence of and death from CRC in LS by the recognition and removal of adenomas and detection of cancer at an earlier stage ^2^. Furthermore, because LS is dominantly inherited, risk increases for family members biologically related to the proband, and every first-degree relative of an affected individual is at 50% risk for carrying the gene mutation and therefore for also having LS. Thus, the identification of an individual with LS has ramifications for both the patient and his or her extended family.

Recognizing LS and individuals at risk can be challenging. The clinical presentation is not always “textbook”, and there tend to be overlapping phenotypes with sporadic CRC and CRC within other hereditary syndromes. As the genetics of this syndrome have become further unveiled in recent years, there have been advances in our understanding of the disease and new concepts in its diagnosis and management. It is difficult to stay abreast of this rapidly changing field. This article discusses some of the recent advances over the last few years in the understanding of LS and how it has impacted clinical management. This is not intended to be a comprehensive review of LS but rather focuses on important recent developments in identification through universal testing of CRCs, risk stratification by site of mutation, improved classification of LS-related phenotypes, implications of extended surgical resection, and new thoughts on chemoprevention. Although LS is a multi-organ disease, the scope of this article is limited mainly to colorectal manifestations and management.

## Genetics and diagnostic approaches

LS is caused by a pathogenic mutation in one of four DNA MMR genes: *MLH1*, *MSH2*, *MSH6*, or *PMS2*. Additionally, there are cases of LS caused by germline deletions of the 3′ end of *EPCAM*, a gene located immediately upstream of *MSH2*, and germline *MLH1* promoter hypermethylation ^3, 4^. Loss of MMR function through one of these mechanisms results in an accumulation of uncorrected mismatches that commonly occur in DNA microsatellite regions (regions of DNA with repetitive elements, including mononucleotides, dinucleotides, and trinucleotides) and eventual initiation of a tumor. Since these errors tend to occur in microsatellite areas, these tumors are called microsatellite-unstable or have high microsatellite instability (MSI). The molecular hallmark of LS cancers is this high MSI, which is present in approximately 93% of tumors ^5^.

The discovery of the genetic basis of the disease has revolutionized LS diagnosis and management. However, controversy exists regarding who should be tested for LS and how it should be done. For affected individuals when tumor tissue is available, testing relies on the characteristics of Lynch-related cancers: high MSI and loss of MMR protein expression. Microsatellite stability or instability is measured by using a tumor DNA polymerase chain reaction-based test that evaluates differential DNA fragment lengths on the basis of the presence of a mutation. The presence or loss of MMR proteins within the tumor can be determined by immunohistochemistry (IHC) on paraffin-embedded tissue for one of the four previously mentioned MMR proteins. The IHC results then can guide formal germline testing for mutations in the specific gene that is not expressed ^6^.

### Selecting patients for testing

Approximately one of every 35 patients diagnosed with CRC will have LS, representing a high prevalence for a highly penetrant, dominantly inherited, potentially lethal condition ^7^. Identifying at-risk patients remains crucial to the mission of preventing cancers and death from cancer. The most efficient and cost-effective way of screening remains debated; however, the cost analysis is evolving rapidly with the falling costs of DNA sequencing.

Traditionally, clinical criteria such as Amsterdam criteria or Bethesda guidelines have been used to identify at-risk patients and thus select those for testing. Amsterdam criteria rely on an accurate and detailed family history and have a sensitivity of less than 50% ^8^. Bethesda guidelines, which incorporate pathology criteria, have a slightly higher sensitivity, approximately 70% ^7^. These guidelines have been shown to be poorly implemented in clinical practice; they have a miss rate of about 28% of MSI/IHC-positive patients. Furthermore, screening only patients younger than 50 years will result in missing about half of LS cases.

In response to the lack of sensitivity, some groups have developed prediction models to help identify at-risk patients. Examples include MMR predict ^9^, MMRpro ^10^, and PREMM ^11^. These models function as a surrogate tool to provide quantitative estimates of the likelihood of an MMR gene mutation. Each model relies on certain clinical criteria to estimate a person’s risk of having LS. The overall sensitivity and specificity are above 90% for these models ^7^, except for the PREMM model, which has a specificity of about 67% ^12^. All models can be accessed and are available for use online. A risk stratification of more than 5% is highly predictive of MMR mutation and justifies pursuing genetic risk assessment. Despite the demonstrated sensitivity of these models, they still rely on clinical parameters, clinician awareness, and patient recall, which are suboptimal and limit their effectiveness. A recent development for identifying at-risk patients for LS is to screen all CRCs. In 2009, the Evaluation of Genomic Applications in Practice and Prevention Working Group recommended that all newly diagnosed CRCs undergo MSI or IHC or both ^13^. These guidelines are endorsed by the Collaborative Group of the Americas on Inherited Colorectal Cancer ^14^. Subsequently, the National Comprehensive Cancer Network (NCCN) further enforced the importance of universal screening but restricted it to those younger than 70 years of age and those older than 70 who met Bethesda guidelines ^15^. In a recent analysis of Cleveland Clinic data, 18% of identified LS cases via the CRC universal screening program were diagnosed in patients older than 70 years (unpublished data). On the basis of this study, the authors support universal screening of all CRC, regardless of age.

Routine screening for LS by using MSI, IHC, and *MLH1* promoter hypermethylation in patients with CRC has been shown to be a cost-effective strategy with important clinical benefits for patients with CRC and their relatives ^16^. CRC surveillance by colonoscopy for mutation carriers decreases development of cancer by more than 60% and decreases mortality from cancer by 72% ^2^. Therefore, the cost of routine screening should be weighed against the benefits of preventing cancers. Studies have used mathematical models to evaluate the cost-effectiveness of universal screening for LS. When at least three at-risk relatives per proband were tested for LS, the cost per life-year gained was approximately $50,000, which is generally accepted for cost-effectiveness at the population level ^17^. As another example, a sample of 150,000 cases of CRC was hypothetically analyzed under the assumption that 2.8% (4,200) of these cases would be LS. With the average person having four first-degree family members (each with a 50% chance of inheriting LS) and eight second-degree relatives (each with a 25% chance of inheriting LS), a total of 16,800 potential individuals with LS would be identified each year. Given that 50% of these individuals (8,400 people) will develop CRC, subsequent enrollment into colonoscopy surveillance programs was associated with a 60% reduction in CRC and prevented 5,040 CRCs ^18^. However, it is estimated that only a small fraction of LS mutation carriers are diagnosed at this time and that an LS screening program would also increase this fraction substantially.

### Selecting a testing algorithm

The exact testing algorithm to be used for screening of a CRC is not definitive. In its recently updated guideline on the diagnosis and management of LS, the American Gastroenterology Association strongly recommended that all CRCs be screened by using MSI or IHC for the expression of MMR proteins ^19^. Tumors that are found to be MSI high or lack MMR protein expression should undergo further testing. If MLH1 protein is lost, the reflex testing should include analysis for a *BRAF* mutation or methylation of the *MLH1* promoter region, as these findings are associated with acquired hypermethylation and do not have an inherited basis for loss of MLH1 expression as seen in LS. If *BRAF* is wild-type or *MLH1* is not hypermethylated (or both occur), then it is recommended to proceed with germline mutation testing of *MLH1*. However, up to 40% of tumors can be *BRAF* wild-type and still have *MLH1* promoter hypermethylation; therefore, in some patients, both tests may be done. If expression of the MMR proteins MSH2, MSH6, or PMS2 is lacking by IHC, then germline testing should commence for the specific gene of the protein lost ^6^.

## Clinical implications of specific gene mutations

The cumulative risk of cancer varies depending on which MMR gene is mutated. Our ability to now classify a family by the precise mutation provides health-care providers with better data to educate patients about individual cancer risks. Patients with a germline mutation in *MLH1* or *MSH2* have an overall higher cancer risk (44–79% and 38–78%, respectively) compared with carriers of *MSH6* and *PMS2* mutations. *MLH1* and *MSH2* mutation carriers have the highest cumulative risk for CRCs at age 70 (50–65% and 40–65%, respectively), and the mean age of onset is 43–46 years ^20^. Patients with *MSH6* mutations tend to develop cancers at a later age. The risk for all LS-related tumors is lower in *MSH6* than *MLH1* and *MSH2* mutation carriers at the age of not more than 50 years (22% versus 40%). Owing to the higher mean age of cancer onset, by the age of 70, the cumulative risks are similar (73% versus 78%). In terms of specific cancer types, male *MSH6* mutation carriers have a CRC cumulative risk at age 70 similar to other genes (69%), but the risk is only 30% for female *MSH6* mutation carriers. However, female *MSH6* mutation carriers have a higher cumulative risk of endometrial cancer (71% versus 27% for *MLH1* and 40% for *MSH2* mutation carriers) ^21^. The penetrance for *PMS2* mutation carriers appears to be lower than that of other MMR gene mutations. At the age of 70, carriers have cumulative risks of 25–30% for LS-related cancers, 15–20% for CRC, and 15% for endometrial cancer ^22^.

These findings have led some groups to recommend different screening and surveillance programs depending on the organ and gene mutation. However, the authors caution against increasing patient age to initiate screening, as we have identified cancers at ages younger than 30 for *PMS2* and *MSH6* gene mutation carriers. In fact, the NCCN has recently retracted its recommendation to start screening patients with these mutations at a later age and has the same recommendations regardless of the causative gene.

## Lynch-like syndrome or Tumor Lynch

The increased use of tumor testing as an LS screening tool has led to some interesting findings. Among patients whose tumors demonstrate MSI and have loss of MMR protein expression, there is a subset of patients who do not have an identifiable germline mutation to confirm the diagnosis of LS. In the past, these patients were traditionally managed as LS because of the reluctance of health-care providers to assume that current genetic testing approaches were perfect and did not miss an underlying genetic cause. However, recent work by several groups has shown that approximately 50% of these cases can be explained by biallelic somatic mutations in the tumor ^23– 25^. Thus, these patients do not have LS and consequently the patient and their families are not at increased risk and do not require advanced screening. Importantly, 50% of these cases of Tumor Lynch are still not defined. In this situation, a family history is even more critical to help assess likely risk. If LS cannot be eliminated from the diagnosis, these patients should be managed as if they have LS, especially if they have a suspicious family history.

## Adenomas in Lynch syndrome

Although LS is traditionally considered to be a non-polyposis syndrome, recent work has defined the adenoma burden in patients with this condition. Forty-one percent of patients with LS had at least one adenoma, including 2% with six to nine adenomas and 4% with more than 10 cumulative adenomas. One patient was found to have 22 synchronous adenomas during a screening colonoscopy ^26^. These findings raise awareness that adenomatous oligopolyposis can exist in LS. If one is suspicious that a patient could have LS, the finding of oligopolyposis should not preclude diagnostic evaluation.

## The utility of extended surgical resection

Since LS is caused by an inherited germline mutation, every cell contains that mutation and the entire colon and rectal epithelia are at risk for developing cancer. Thus, when a CRC is found, consideration must be given to extended surgical resection which is prophylactic against forming metachronous cancers in any remaining colon or rectum. For example, if a right colon cancer is diagnosed in a patient with LS, a total abdominal colectomy with an ileorectal anastomosis (rather than a right colectomy) is recommended. Multiple studies in the last few years have supported decreased metachronous CRC following extended resection. The Cleveland Clinic group reported metachronous CRC in 25% of putative Lynch patients undergoing segmental resection compared with 8% after total abdominal colectomy ^27^. Importantly, this study also reported on the identification and removal of high-risk adenomas during surveillance in this population. As these lesions have a high likelihood of progression to cancer if not removed, they are considered surrogates for cancer. Another report demonstrated higher rates of metachronous CRC in patients with limited/segmental resections (26%) as opposed to extended/prophylactic resection (6%), confirming the advantages of extended resections ^28^. A retrospective study using the Colon Cancer Family Registry aimed to evaluate the risk of developing metachronous CRC in LS patients who had either segmental or extensive (subtotal or total) resection in their index surgery. Twenty-two percent of patients undergoing segmental resection developed a metachronous CRC, and the rates of cumulative projected risk were 16%, 41%, and 62% at 10, 20, and 30 years, respectively ^29^.

It is important to note that these data are all from retrospective studies and there are no prospective trials that prove extended resection improves overall survival. Similarly, there are no trials that demonstrate extended resection is superior to close colonoscopic surveillance after segmental resection. However, there are challenges to a successful postoperative surveillance regimen. Patient compliance, suboptimal bowel preparation, endoscopist skill, and the biology of LS-associated adenomas and cancers ^30, 31^ all contribute to the development of interval cancers. Interval cancers still develop in 35% of cases under surveillance ^32, 33^. Thus, the authors favor extended resection in the medically fit patient.

Obviously, the ultimate decision regarding the extent of resection is determined after consideration of the risks and benefits for each individual patient. Extended resection does result in more frequent bowel movements but also in similar quality of life ^34– 36^. The preoperative discussion should focus on the oncologic benefits and expected bowel function. Patient factors to consider include age, sphincter function, medical comorbidities, willingness to comply with surveillance, and patients’ wishes. For example, an elderly patient with multiple comorbidities that may limit life expectancy might be better served by a less extensive segmental resection followed by close surveillance. A similar line of reasoning exists for patients with stage IV disease that requires resection of the primary. In this situation, they are more likely to succumb to metastatic disease rather than a metachronous CRC and a segmental resection is more prudent.

## Chemoprevention

The routine use of chemopreventive agents for patients with LS remains debated. The Colorectal Adenoma/Carcinoma Prevention Program (CAPP) has been dedicated to studying the chemoprevention of CRC. CAPP-2 is the only randomized placebo-controlled trial for patients with LS. The trial included 937 patients who were randomly assigned to receive resistant starch, 600 mg aspirin, 600 mg aspirin plus resistant starch, or 600 mg aspirin plus placebo ^37^. The initial findings did not show any difference in colorectal adenoma or cancer formation up to 4 years. However, analysis at a longer follow up revealed that patients who took aspirin for at least 2 years had a lower incidence of CRC and LS-related cancers than those who took placebo at follow up of nearly 56 months ^38^.

It is notable that aspirin is not available in the United States in 600 mg doses. The exact dose, duration of use, and associated side effects need to be further evaluated before recommending routine use in patients with LS. There is an ongoing CAPP-3 trial which will evaluate the effects of three different doses of aspirin (100 mg, 300 mg, and 600 mg) taken for 5 years. The targeted enrollment worldwide is 3,000 patients with LS.

## Future directions

As the basic science of hereditary CRC syndromes continues to be unraveled, the field is rapidly changing. The development of next-generation sequencing gene panel testing will improve the ability to diagnose more patients and to define exact mutations. Multiple commercial tests are readily available and their use is increasing. Panel testing should be ordered and interpreted in the context of genetic counseling, as interpretation and appropriate application of the results are critical. The ability to elucidate a precise genetic diagnosis for LS allows a better understanding of its natural history. There are multiple international collaborative groups that are building clinical and genetic databases for patients with LS. Examples include the International Mismatch Repair Consortium and the Colon Cancer Family Registries. Through these collaborative efforts, the nuances between different gene mutations, the relevance of variants of unknown significance, and the spectrum of the syndrome will be better delineated. Furthermore, collaborative groups with their family registries can serve as the foundation for prospective studies to discover more accurate or efficient screening protocols, to study new chemoprevention agents, and to identify non-genetic modifiers that may influence the clinical phenotype.
